# Fecal Microbiota Transplantation Attenuates Inflammation Through TGF-β1/Smad Signaling Pathway in Caco-2 and RAW264.7 Cells

**DOI:** 10.5152/tjg.2025.25172

**Published:** 2025-10-10

**Authors:** Jinlang Qiu, Caixian Wu, Sheng Li, Xiaoyu Yang

**Affiliations:** 1 Department of Clinical Laboratory, Fuzhou Hospital of Traditional Chinese Medicine, Fujian, China; 2 Department of Anus-Intestines, The Second Affiliated Hospital of Fujian University of Traditional Chinese Medicine, Fujian, China; 3 Department of Medical Oncology, Fuzhou Hospital of Traditional Chinese Medicine, Fujian, China; 4 Fuzhou Hospital of Traditional Chinese Medicine, Fujian, China

**Keywords:** Fecal microbiota transplantation, lentivirus packaging, Smad signaling pathway, TGF-β1, ulcerative colitis

## Abstract

**Background/Aims:**

Ulcerative colitis (UC) represents a persistent inflammatory condition that influences millions of people worldwide, with rising prevalence and limited treatment options. Current therapies, such as corticosteroids and immunosuppressants, offer symptom relief but are associated with significant adverse effects. Fecal microbiota transplantation (FMT) is being increasingly viewed as an effective alternative, but the molecular basis for its benefits in UC is still not fully understood. This study aimed to explore the function of the transforming growth factor-beta 1 (TGF-β1)/Smad signaling cascade in FMT-induced remission of UC.

**Materials and Methods:**

Stable Smad3-knockdown and Smad3-overexpression Caco2 cell lines were established via lentivirus-mediated transduction. These modified Caco-2 cells were co-incubated with RAW264.7 macrophages to mimic intestinal inflammation in vitro. Following FMT treatment, the expression of major components of the TGF-β1/Smad signaling cascade was assessed.

**Results:**

The results demonstrated that FMT markedly downregulated TGF-β1, Smad2, and Smad3 expression, while enhancing Smad7 expression in both Smad3-knockdown and overexpression cell lines. In addition, FMT treatment attenuated the phosphorylation of Smad2 and Smad3, indicating a decrease in the activation of the TGF-β1/Smad signaling pathway.

**Conclusion:**

These findings show that by optimizing FMT treatments to focus on the specific pathway, an improvement of therapeutic outcomes can be achived for UC patients.

**Main Points**

- Fecal microbiota transplantation (FMT) significantly downregulated pro-inflammatory transforming growth factor-beta 1 (TGF-β1), Smad2, and Smad3 expression while upregulating the anti-inflammatory Smad7 in both Smad3-knockdown and Smad3-overexpression Caco-2 cells.
- FMT reduced Smad2 and Smad3 phosphorylation, indicating effective suppression of TGF-β1/Smad pathway activation in an in vitro model of ulcerative colitis (UC).
- Co-treatment with the TGF-β1 inhibitor SB431542 synergistically enhanced FMT’s suppression of Smad signaling and further promoted Smad7 expression.
- Lentiviral-mediated stable modulation of Smad3 provided a robust framework to dissect the mechanistic influence of TGF-β1/Smad signaling pathway in FMT-induced immune regulation.
- These findings highlight TGF-β1/Smad pathway modulation as an essential mechanism underlying FMT’s anti-inflammatory effect, supporting its potential as a novel therapeutic strategy for UC.

## Introduction

Ulcerative colitis (UC) constitutes a persistent inflammation of the colon and rectum, with a global prevalence estimated at 5 million cases and a growing incidence.^1^ Although current therapies such as corticosteroids and immunosuppressants provide symptom relief, their prolonged administration frequently leads to notable adverse effects, and many individuals experience disease relapse.^2^ Therefore, there is a growing need for alternative therapeutic strategies with fewer adverse effects. Fecal microbiota transplantation (FMT) has shown promise as an intervention capable of addressing UC by modulating gut microbiota and alleviating inflammation.^3^ However, the exact molecular mechanisms by which FMT induces remission in UC remain poorly understood.

Emerging evidence has highlighted that the transforming growth factor-beta 1 (TGF-β1)/small mothers against decapentaplegic (Smad) pathway is implicated in the pathogenesis of UC.^4^^-^^8^ This pathway is pivotal in modulating immune responses and upholding intestinal homeostasis. Dysregulation of TGF-β1/Smad signaling is considered a contributor to chronic inflammation and structural damage in UC.^9^ The previous study demonstrated that FMT can suppress the TGF-β1/Smad signaling cascade, leading to reduced inflammatory responses in experimental models of inflammatory bowel disease (IBD).^10^ However, the specific role of this pathway in FMT-mediated modulation of UC remains unclear and warrants further investigation.

In this study, the aim was to elucidate the role of the TGF-β1/Smad signaling pathway in FMT-induced remission of UC. To achieve this, short interfering RNAs (siRNAs) targeting Smad3 were designed, an essential modulator of the TGF-β1/Smad signaling cascade. These siRNAs were used to knock down Smad3 expression in Caco-2 cells, which were subsequently co-incubated with RAW264.7 macrophages to replicate inflammatory bowel conditions in vitro. By analyzing the expression levels of key elements within the TGF-β1/Smad pathway in FMT-treated cells, it was sought to uncover the molecular mechanisms underlying FMT-mediated modulation in UC.

## Materials and Methods

### Preparation of Fecal Microbiota Supernatant

Fecal microbiota transplantation was obtained from healthy male SPF-grade C57BL/6 mice (5 weeks old, 15-17 g, n = 20), sourced from the Experimental Animal Center of Yangzhou University. All animals resided in a barrier facility under specific pathogen-free (SPF) protocols, incorporating a controlled thermal range of 22 ± 2°C, humidity control between 50% and 60%, air exchange rate of 10-15 cycles/hour, and alternating 12-hour light/darkness illumination. Mice had access to autoclaved drinking water and sterilized standard pellet feed ad libitum. No antibiotics or experimental treatments were administered prior to fecal collection, and all animals were confirmed to be in good health. Fresh fecal pellets were collected under sterile conditions within 30 minutes of spontaneous defecation. A total of 7 g of pooled fecal samples was suspended in 15 mL of sterile 0.9% normal saline by vortex mixing. The fecal suspension was then filtered through a stainless-steel mesh to remove large particles and centrifuged at 6000 × g for 15 minutes. The supernatant was retained and cooled at 4°C for subsequent use in in vitro co-culture experiments. All procedures were conducted aseptically to preserve microbial viability. All in vivo experiments were reviewed and approved by the Animal Ethics Committee of Yangzhou University, in compliance with national guidelines concerning the proper care and ethical use of lab animals (Approval reference: 202309017, date: September 11, 2023)

### Culture Conditions for Cell Lines

Human embryonic kidney 293T cells (HEK 293T cells, ATCC CRL-3216) were propagated in Dulbecco’s modified Eagle’s medium (DMEM, No. 06-1055-57-1ACS, Biological Industries, Kibbutz Beit Haemek, Israel) containing 20% fetal bovine serum (FBS, No. FSP500, ExCell Bio Co., Ltd., Suzhou, China) and 1% penicillin/streptomycin. Human colon adenocarcinoma cell line Caco-2 (ATCC HTB-37) and mouse monocyte-macrophage leukemia cells (RAW 264.7, ATCC TIB-71TM) were cultured in Minimum Essential Medium (MEM, No. PM150410, Procell Life Science & Technology Co., Ltd., Wuhan, China), enriched with 20% FBS and 1% antibiotic mixture. Cells were kept at 37°C in 5% CO_2_-supplemented humidified incubators.

### SiRNA Design and Screening

Four short interfering RNAs (siRNAs) targeting Smad3 were designed using BLOCK-iT™ RNAi Designer (https://rnaidesigner.thermofisher.com/rnaiexpress/) and synthesized by Suzhou Genepharma Co., Ltd. The sequences of the siRNAs are presented in Table 1.

**Figure 1.**
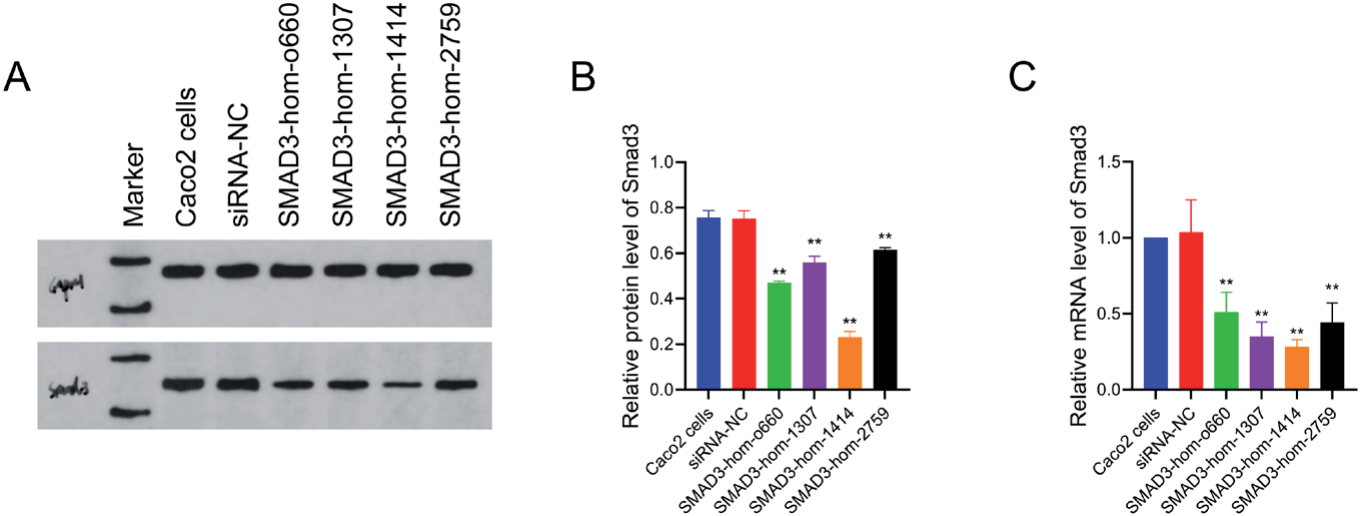
Screening of siRNA targeting Smad3. (A) Western blot of Smad3 and Glyceraldehyde-3-phosphate dehydrogenase(GAPDH) (loading control) protein levels in Caco-2 cells transfected with 4 siRNAs targeting smad3 and a negative control siRNA (siRNA-NC). (B) Quantification of Smad3 protein levels. (C) High-resolution melting quantitative reverse transcription polymerase chain reaction analysis of Smad3 mRNA levels in Caco-2 cells transfected with 4 siRNAs targeting smad3 and a negative control. Data were presented as mean ± SD (n = 3). ^**^P < .01 vs. siRNA-NC.

Caco-2 cells were plated (5.0 × 10^5^ per well) in 6-well plates and incubated for 24 hours. Subsequently, 50 nM siRNA was introduced via Lipofectamine® 2000 (Thermo Fisher, Waltham, MA, USA) following the supplier’s protocol. Post-transfection, the efficiency of Smad3 silencing was assessed using Western blot and quantitative reverse transcription polymerase chain reaction (RT-PCR). FAM-siRNA-transfected Caco-2 cells functioned as a control group. Figure 1 shows the expression levels of Smad3 protein and mRNA after transfection with these siRNAs, illustrating the efficiency of Smad3 silencing in Caco-2 cells.

### Plasmids Construction

**Figure 2.**
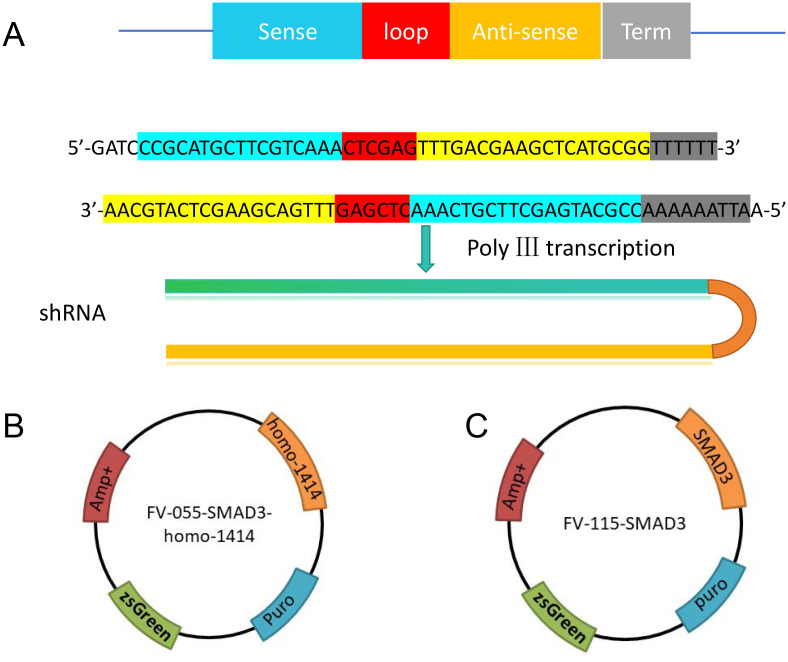
Construction of Smad3-knockdown and Smad3-overexpression plasmids. (A) Schematic representation of the shRNA design. (B) Construction of the Smad3-knockdown plasmid FV055-shRNA. (C) Construction of the Smad3-overexpression plasmid FV115-Sm3. shRNA refers to short hairpin RNA used for gene silencing.

Plasmids FV055 (Smad3 knockdown) and FV115 (Smad3 overexpression) were previously stored in the laboratory. The short hairpin RNA (shRNA) targeting SMAD3-homo-1414 was designed as described in a previous study^11^ and cloned into the FV055 plasmid (Figure 2A). The *Smad3* gene was cloned via amplification and ligated into the FV115 plasmid to create an overexpression plasmid. All plasmids were validated by DNA sequencing and fluorescence microscopy to confirm correct insertion.

### Lentivirus Packaging and Stable Cell Line Selection

HEK 293T cells were seeded in 10 cm^2^ Petri dishes and allowed to adhere for 24 hours. At 70% confluence, the cells were transfected with 10 µg of either FV055-shRNA or FV115-Smad3 expression plasmids, together with 5 µg each of psPAX2 and pMD2G helper vectors. The culture medium was changed once after 24 hours. Supernatants enriched with lentiviral particles were harvested at both 24 hours and 48 hours following transfection, then filtered and concentrated. For transduction, Caco-2 cells were plated into 12-well plates 24 hours before viral infection. Lentivirus (10 µL, MOI = 50) was then added, and puromycin at 5 μg/mL was applied for stable integrants after 48 h. The expression of green fluorescent protein (GFP) was used to identify successfully transduced cells.

### Establishment of an In Vitro Co-Culture Model Using Caco-2 and RAW264.7 Lines

Smad3 knockdown, Smad3 overexpression, or control Caco-2 cells were grown alongside RAW264.7 cells using a Transwell insert system (Corning, NY, USA) to imitate intestinal inflammation conditions in vitro.^12^ A seeding density of 3.75 × 10^5^ Caco-2 cells was used in Transwell inserts, and 8.5 × 10^5^ RAW264.7 cells were added to 6-well plates. The 2 cell lines were pre-incubated for 24 hours to support adhesion. At the 24-hour mark, cells were treated with 100 MOI/0.5 mL FMT, 10 μmol/L prednisone, culture medium, or 100 MOI/0.5 mL FMT + 10 μmol/L SB431542 (TGF-β1 inhibitor) for 6 hours. Medium changes were performed every 2 to 3 days to maintain favorable conditions for cell growth and differentiation, with cells used between passages 48 and 62 for Caco-2 cells and passages 10 and 30 for RAW264.7 cells.

### Experimental Grouping

The experimental groups were as follows: Blank (normal Caco-2 cells), KD+FMT (Smad3 knockdown Caco-2 cells treated with FMT), KD+Prednisone (Smad3 knockdown Caco-2 cells treated with prednisone), KD+culture medium (Smad3 knockdown Caco-2 cells treated with culture medium), KD+FMT+SB431542 (Smad3 knockdown Caco-2 cells treated with FMT and TGF-β1 inhibitor SB431542), OE+FMT (Smad3 overexpression Caco-2 cells treated with FMT), OE+Prednisone (Smad3 overexpression Caco-2 cells treated with prednisone), OE+culture medium (Smad3 overexpression Caco-2 cells treated with culture medium), and OE+FMT+SB431542 (Smad3 overexpression Caco-2 cells treated with FMT and SB431542).

### Western Blotting

Cell lysis was carried out using radio immuno precipitation assay (RIPA) buffer (No. MA0151, MeilunBio Co., Ltd., Dalian, China), and protein concentration was quantified via a BCA-based colorimetric assay (No. G3422, GBCBIO Technologies, Guangzhou, China). Protein lysates underwent separation via 10% SDS-PAGE, and polyvinylidene fluoride (PVDF) membranes were used for blotting. Blocking was done with 5% milk for 1 hour at ambient temperature, followed by overnight incubation in primary antibodies at 4°C. Triple washes in 0.1% Tween-20-containing Tris-HCl buffer were followed by incubation with horseradish peroxidase-conjugated secondary antibodies (goat anti-rabbit IgG: No. BA1054, 1:2000; goat anti-mouse IgG: No. BA1051, 1:10000; Boster Biological Technology Co. Ltd., Wuhan, China) for 1 hour at room temperature. Protein bands were detected through enhanced chemiluminescence (ECL Plus), and their intensities were quantified with ImageJ, with GAPDH used to normalize protein expression.

The following primary antibodies were used: anti-TGF-β1 (No. AF1027, 1:1000, Affinity, Cincinnati, USA), anti-Smad2 (No. 12570-1-ap, 1:5000, Proteintech Group, Wuhan, China), anti-phosphorylated Smad2 (No. AP1007, 1:1000, ABclonal Technology Co., Ltd, Wuhan, China), anti-Smad3 (1:5000, No. 66516-1-ig, Proteintech Group, Wuhan, China), anti-phosphorylated Smad3 (1:1000, No. AP0827, ABclonal Technology Co., Ltd., Wuhan, China), anti-Smad4 (No. 10231-1-ap, 1:1000, Proteintech Group, Wuhan, China), anti-Smad7 (No. 25840-1-ap, 1:5000, Proteintech Group, Wuhan, China), and anti-GAPDH (No. AB-P-R 001, 1:1000, Goodhere Biotech Co., Hangzhou, China).

### High-Resolution Melting Quantitative Reverse Transcription Polymerase Chain Reaction

Caco-2 cell RNA extraction was conducted using TRIzol reagent (No. 15596-026, Invitrogen, CA, USA). cDNA synthesis was achieved via HiScript® II Q RT SuperMix for qPCR (+gDNA wiper) (No. R223-01, Vazyme Biotech Co., Ltd., Nanjing, China) according to the supplier’s manual. Amplification involved SYBR Green Master Mix (No. Q111-02, Vazyme Biotech Co., Ltd., Nanjing, China) and Taq Plus DNA Polymerase (No. ET105-02, Tiangen Biotech (Beijing) Co., Ltd, Beijing, China). Polymerase chain reaction primers for TGF-β1/Smad signaling pathway components are shown in Table 2. The PCR conditions were as follows: predenaturation at 94°C (10 minutes), denaturation at 95°C (15 seconds), and annealing at 60°C for 1 minute (×40). Melting curve steps were set to 95°C/15 s, 60°C/60 s, and 95°C/15 s. Quantitative analysis was performed on the QuantStudio 1 (Applied Biosystems/ThermoFisher, USA).

### Statistical Analysis

SPSS version 19.0 (IBM SPSS Corp.; Armonk, NY, USA) was utilized for statistical evaluation. Shapiro-Wilk tests assessed the distribution of continuous data, and Levene’s test was used to examine variance equality. Data conforming to normality were reported as mean ± SD and median (Q1, Q3) for skewed distributions. Between-group differences were evaluated by one-way ANOVA with Tukey’s post hoc test or Mann–Whitney *U* for data not meeting parametric assumptions. *P* < .05 was considered indicative of statistical significance.

## Results

### Smad 3 Silencing Efficiency of Various siRNAs

To evaluate the efficiency of Smad3 silencing, Caco-2 cells were transfected with 4 siRNAs targeting Smad3 and a negative control (SMAD3-NC), followed by Western blotting and high-resolution melting quantitative RT-PCR. As shown in Figure 1A and B, the relative protein levels of Smad3, normalized to GAPDH, were 0.465, 0.539, 0.249, 0.609, and 0.776 for SMAD3-homo-660, SMAD3-homo-1307, SMAD3-homo-1414, SMAD3-homo-2759, and SMAD3-NC–transfected cells, respectively. The SMAD3-homo-1414 group exhibited the lowest Smad3 protein expression. Similarly, Figure 1C illustrates the relative mRNA levels of Smad3 in the same groups, showing values of 0.512, 0.350, 0.284, 0.445, and 1.039 for SMAD3-homo-660, SMAD3-homo-1307, SMAD3-homo-1414, SMAD3-homo-2759, and SMAD3-NC–transfected cells, respectively. The SMAD3-homo-1414 group also exhibited the lowest Smad3 mRNA expression among all siRNA-transfected cells. Given that SMAD3-homo-1414 achieved the most efficient silencing of Smad3 at the transcript and translation levels, this sequence was used for further Smad3 knockdown studies.

### Effect of Fecal Microbiota Transplantation on Transforming Growth Factor-Beta 1/Smad Signal Transduction

To explore the effect of FMT on the TGF-β1/Smad pathway, stable Smad3-knockdown and Smad3-overexpression Caco2 cell lines were successfully established using lentivirus packaging (Figure 2) and cultured together with RAW264.7 macrophages to mimic intestinal inflammation in vitro.

**Figure 3.**
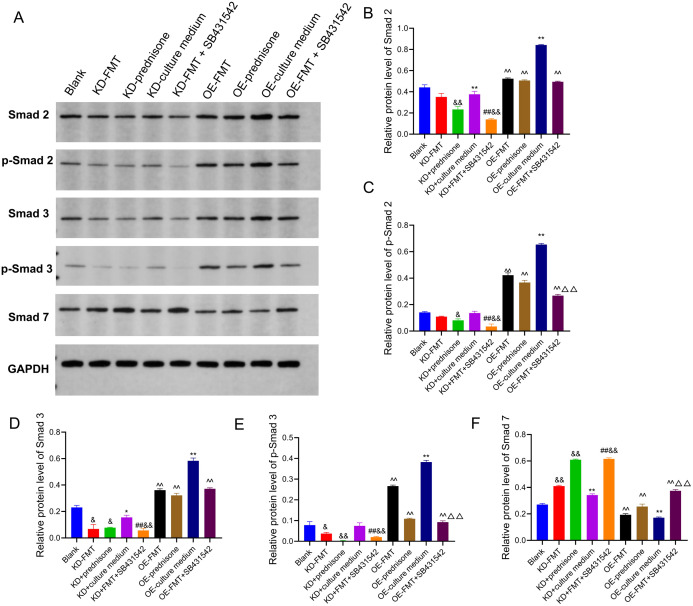
Western blot analysis of TGF-β1/Smad signaling components in Smad3-knockdown and Smad3-overexpression Caco-2 cells. (A) Western blot analysis of Smad2, p-Smad2, Smad3, p-Smad3, Smad7, and GAPDH (loading control) in modified Caco-2 cells. Line 1: Blank; Line 2: KD+FMT; Line 3: KD+prednisone; Line 4: KD+culture medium; Line 5: KD+FMT+SB431542; Line 6: OE+FMT; Line 7: OE+prednisone; Line 8: OE+culture medium; Line 9: OE+FMT+SB431542. (B) Relative protein levels of Smad2. (C) Relative protein levels of p-Smad2. (D) Relative protein levels of Smad3. (E) Relative protein levels of p-Smad3. (F) Relative protein levels of Smad7. Data were presented as mean ± SD (n = 3). ^*^P < .05, ^**^P < .01 vs. Blank; ^##^*P* < .01 KD+FMT; ^&^*P* < .05, ^&&^*P* < .01 vs. KD+Culture medium; ^^^^*P* < .01 vs. OE+Culture medium; ^△△^*P* < .01 vs. OE-FMT. p-Smad: phosphorylated Smad proteins.

Western blot analysis (Figure 3) revealed significant alterations of TGF-β1/Smad pathway–related core proteins in response to FMT and prednisone treatment. In Smad3-knockdown Caco-2 cells, FMT treatment significantly downregulated both Smad3 and p-Smad3 protein levels (*P* < .05), while Smad7 protein expression was significantly upregulated (*P* < .01). The prednisone-treated group showed similar effects, with a significant decrease of Smad2/p-Smad2 and Smad3/p-Smad3, accompanied by a significant increase in Smad7 expression (*P* < .05 or *P* < .01). In Smad3-overexpression Caco-2 cells, FMT significantly downregulated Smad2/p-Smad2 and Smad3/p-Smad3 protein levels, while Smad7 was also markedly upregulated (all *P* < .01).

**Figure 4.**
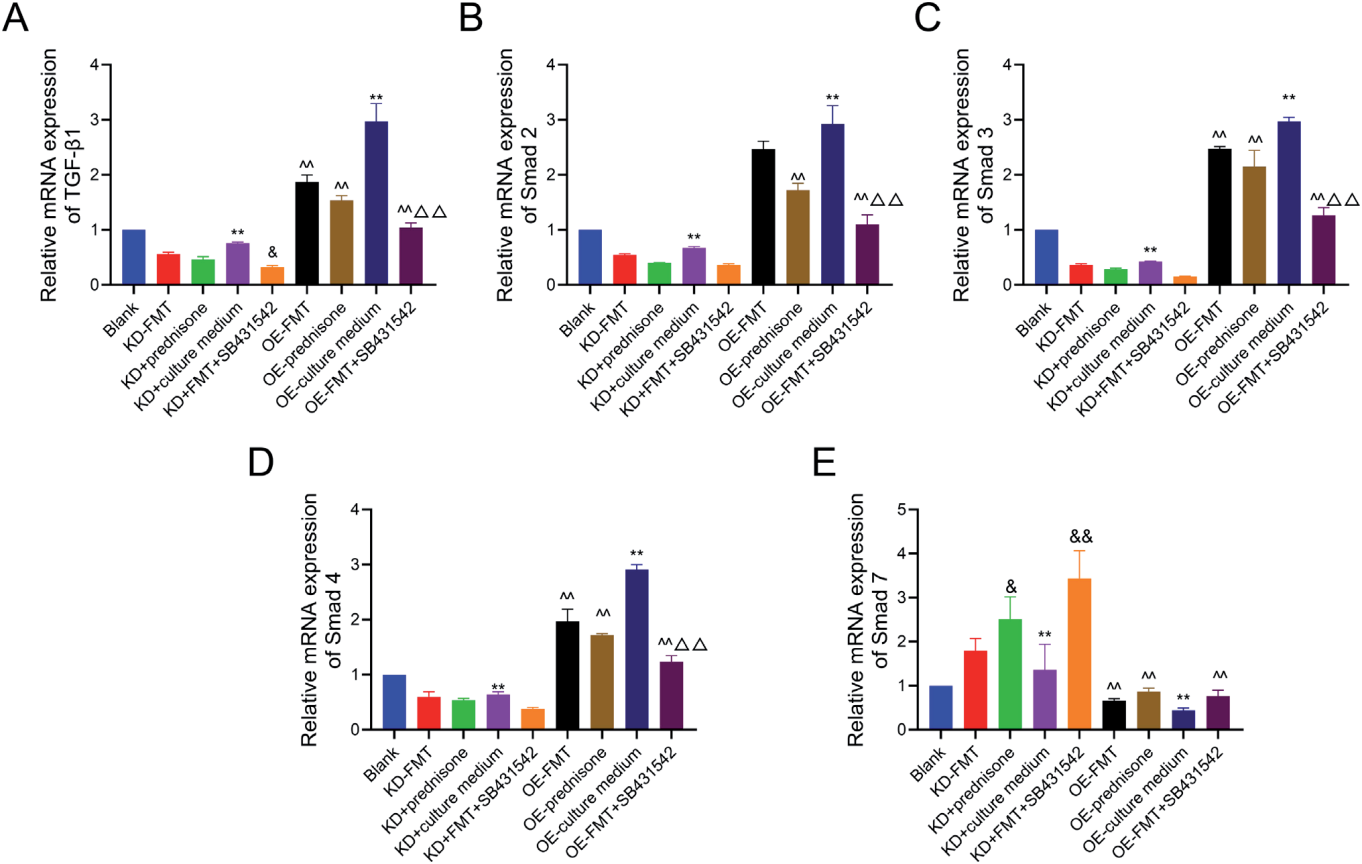
High-resolution melting quantitative reverse transcription polymerase chain reaction analysis of transforming growth factor-beta 1/Smad signaling components in Smad3-knockdown and Smad3-overexpression Caco-2 cells. (A) Transforming growth factor-beta 1 mRNA expression in modified Caco2 cells. (B) Smad2 mRNA expression in modified Caco2 cells. (C) Smad3 mRNA expression in modified Caco2 cells. (D) Smad4 mRNA expression in modified Caco2 cells. (E) Smad7 mRNA expression in modified Caco2 cells. Data were presented as mean ± SD (n = 3). ^**^P < .01 vs. Blank; ^&^*P* < .05, ^&&^*P* < .01 vs. KD+Culture medium; ^^^^*P* < .01 vs. OE+Culture medium; ^△△^*P* < .01 vs. OE-FMT.

In addition, quantitative RT-PCR analysis (Figure 4) depicted that FMT exposure led to a marked reduction in TGF-β1, Smad3, and Smad4 transcripts in Smad3-overexpression Caco-2 cells. Conversely, Smad7 transcript levels were significantly upregulated in response to FMT treatment. Prednisone treatment exhibited similar effects, significantly downregulating TGF-β1, Smad2, Smad3, and Smad4 mRNA levels in Smad3-overexpression Caco-2 cells and upregulating Smad7 mRNA levels in both modified Caco-2 cells in both modified Caco-2 cells.

These findings indicate that FMT could modulate the TGF-β1/Smad pathway by suppressing pro-inflammatory Smad proteins and upregulating Smad7, thereby potentially alleviating inflammation, similar to prednisone.

### Combined Effects of Fecal Microbiota Transplantation and SB431542 on Transforming Growth Factor-Beta 1/Smad Signaling

Interestingly, when cells were treated with SB431542, a TGF-β1 inhibitor, in combination with FMT, the changes in protein and mRNA expression were significantly enhanced. In both modified Caco-2 cells, the protein expression of Smad2, p-Smad2, Smad3, and p-Smad3 was further suppressed, and Smad7 expression was more strongly upregulated (Figure 3). Reverse transcription polymerase chain reaction analysis confirmed that SB431542+FMT treatment significantly reduced TGF-β1, Smad2, Smad3, and Smad4 mRNA (*P* < .05 or *P* < .01), further enhancing the anti-inflammatory response (Figure 4).

These findings suggest that SB431542 potentiates FMT’s effects by enhancing the suppression of pro-inflammatory Smad proteins and promoting Smad7 expression, suggesting a synergistic anti-inflammatory action.

## Discussion

Ulcerative colitis (UC) represents a relapsing–remitting subtype of IBD, driven by a complex interplay involving genetic, immune, and microbial factors.^13^ Despite advances in pharmacological treatments, many UC patients fail to achieve long-term remission, highlighting the need for novel therapeutic approaches. Fecal microbiota transplantation has gained increasing attention as a promising therapeutic approach for UC due to its ability to restore gut microbial homeostasis and modulate immune responses.^12^^,^^14^ Previous research has validated the utility of FMT in UC treatment,^15^^-^^18^ with a meta-analysis of 13 randomized controlled trials showing a clinical remission rate of 50.17% in FMT-treated patients, compared to 29.02% in the control group.^19^ Nevertheless, the molecular pathways responsible for FMT’s beneficial outcomes in UC are still poorly understood.

Transforming growth factor-beta 1 plays a central role in regulating intestinal immunity by suppressing inflammation and promoting immune tolerance.^20^^-^^22^ Dysregulation of this pathway, particularly Smad3, is closely associated with the pathogenesis of IBD, comprising UC, where impaired TGF-β1 signaling leads to uncontrolled intestinal inflammation.^23^ Previous studies, including the authors’ own, have shown that therapies targeting the restoration of TGF-β1 signaling hold promise for managing IBD. Notably, in the earlier research, it had been demonstrated that FMT inhibited TGF-β1/Smad signaling to reduce colonic inflammation in IBD rat models. In the present study, these findings were built on by investigating how FMT modulates this signaling cascade at the cellular level, specifically in Smad3-knockdown and Smad3-overexpression Caco-2 cells.

The findings revealed that FMT treatment downregulated pro-inflammatory Smad (Smad2/p-Smad2 and Smad3/p-Smad3) protein expression while simultaneously upregulating Smad7 expression, both at the protein and mRNA levels in modified Caco-2 cells. This indicates that FMT modulates the TGF-β1/Smad signaling cascade by inhibiting the pro-inflammatory Smad proteins, which contribute to the propagation of inflammation, and promoting Smad7, a known inhibitor of TGF-β1 signaling that dampens excessive immune responses.^24^ Interestingly, the potential synergistic effects of combining FMT with the TGF-β1 inhibitor SB431542 were further explored. The results indicated that the addition of SB431542 enhanced the effects of FMT, leading to further suppression of Smad3 and p-Smad3 while promoting a more pronounced upregulation of Smad7. These findings provide strong evidence that FMT may alleviate inflammation in UC through modulation of the TGF-β1/Smad pathway, supporting its therapeutic potential in managing UC.

A critical aspect of this study was the use of RNA interference (RNAi) technology, a commonly employed technique for gene silencing and validation under various conditions,^25^ aimed at elucidating the specific function of Smad3 within TGF-β1/Smad signaling in UC. RNAi techniques have evolved over time with the development of various methodologies, including short interfering RNA, short hairpin RNA, small molecule ribonucleic acid, and others.^11^^,^^26^ In this study, 4 siRNAs were designed targeting Smad3, with Smad3-homo-1414 exhibiting the highest silencing efficiency. Lentiviral-mediated transduction was used to establish stable Smad3-knockdown and Smad3-overexpression Caco-2 cells, which provided valuable tools for studying the impact of Smad3 modulation on UC.^27^ The lentivirus-based system enabled one to achieve stable and consistent gene expression changes, making it an ideal method for examining the effects of Smad3 modulation in UC.

However, while the Caco-2 + RAW264.7 cell model offers valuable insights, it has limitations. The Caco-2 cells, derived from human colon adenocarcinoma, form tight junctions mimicking the intestinal epithelial barrier, which is often compromised in UC.^28^ RAW264.7 macrophages are suitable for studying innate immune functions, given their ability to release TNF-α, IL-6, and IL-1β—key mediators in UC.^29^ However, this model lacks a mucus layer, crucial for impeding microbial penetration and epithelial injury, limiting its ability to fully replicate the in vivo environment, particularly in UC, where the mucus barrier is disrupted. Additionally, the model does not include adaptive immune components like T and B lymphocytes, which are vital in UC’s immune responses. To address these limitations, future studies should incorporate human intestinal organoids and mouse models of UC. Organoids better replicate the intestinal environment, including the mucus layer, and provide a more accurate model of epithelial function and inflammation in UC.^30^ Mouse models of UC, such as those induced by dextran sulfate sodium treatment, allow for the study of both early and late immune responses, enhancing the overall understanding of UC pathophysiology.^31^ These models will complement the Caco-2 + RAW264.7 system and enable deeper investigation of FMT’s therapeutic effects in UC.

The research offers a novel mechanistic understanding of FMT-mediated remission in UC by elucidating its role in modulating the TGF-β1/Smad pathway. Furthermore, it explores the synergistic effects of combining FMT with TGF-β1 inhibitor SB431542, demonstrating enhanced anti-inflammatory effects and offering a potential strategy to improve FMT efficacy in UC treatment.

Despite these significant findings, there are some limitations that should be addressed in future research. First, it relies on in vitro models, and further validation in animal models or clinical studies is needed to confirm its translational relevance. Second, while it has been demonstrated that FMT influences the TGF-β1/Smad signaling, the specific microbial species responsible for this effect remain unidentified; future studies using high-throughput sequencing and microbiome analysis are warranted. Lastly, the long-term efficacy and stability of FMT-induced immune modulation require further investigation to determine its potential for sustained UC remission.

Importantly, FMT is not yet included in current UC treatment guidelines. However, recent clinical studies suggest that FMT may induce remission in some patients with active UC, especially those unresponsive to standard therapies.^3^ These results, showing anti-inflammatory effects comparable to prednisone, support the potential of FMT as an alternative or adjunct to steroid treatment. In addition, the observed synergy between FMT and TGF-β1 inhibition provides a novel therapeutic strategy worth exploring in future clinical studies.

In summary, this study elucidated the role of the TGF-β1/Smad pathway as a key target for FMT-mediated remission in UC. By modulating pro-inflammatory Smad proteins and enhancing Smad7 expression, FMT may offer a novel therapeutic approach for UC patients. Future research focusing on microbiota identification, in vivo validation, and long-term effects will be essential to translate these findings into clinical practice.

This research reveals that FMT reduces inflammation in UC by modulating the TGF-β1/Smad axis in Smad3-knockdown and Smad3-overexpression Caco-2 cells. Such results broaden the comprehension of FMT’s immunomodulatory mechanisms while laying a scientific foundation for future research to optimize its clinical application in UC treatment.

## Figures and Tables

**Table 1.** The Sequences of siRNA Targeting Smad 3

shRNA Name	Sense/Antisense	Sequence (5’-3’)
SMAD3-homo-660	Sense	GCGUGAAUCCCUACCACUATT
	Antisense	UAGUGGUAGGGAUUCACGCTT
SMAD3-homo-1307	Sense	GGAUGCAACCUGAAGAUCUTT
	Antisense	AGAUCUUCAGGUUGCAUCCTT
SMAD3-homo-1414	Sense	CCGCAUGAGCUUCGUCAAATT
	Antisense	UUUGACGAAGCUCAUGCGGTT
SMAD3-homo-2759	Sense	GGCCAACAUAGGCAAAUGATT
	Antisense	UCAUUUGCCUAUGUUGGCCTT
SMAD3-NC	Sense	UUCUCCGAACGUGUCACGUTT
	Antisense	ACGUGACACGUUCGGAGAATT

SMAD3-NC, the negative control targeting non-specific sequences.

**Table 2.** Nucleotide Sequence of Primers Used in Real-Time Polymerase Chain Reaction

Gene	Primer	Sequence (5’-3’)	PCR Product
*SMAD3*	Forward	ACGACTACAGCCATTCCATCCC	128 bp
	Reverse	CATCTGGTGGTCACTGGTTTCTC	
*TGF-β1*	Forward	AACCCACAACGAAATCTATGAC	205 bp
	Reverse	GCTGAGGTATCGCCAGGAAT	
*SMAD2*	Forward	CTTGATGGTCGTCTCCAGGTA	248 bp
	Reverse	AGAGGCGGAAGTTCTGTTAGG	
*SMAD4*	Forward	CTTAGACAGAGAAGCTGGGCG	293 bp
	Reverse	GTCCCCAGCCTTTCACAAAAC	
*SMAD7*	Forward	CTCGGAAGTCAAGAGGCTGTG	137 bp
	Reverse	CCATCGGGTATCTGGAGTAAGG	
*GAPDH*	Forward	TCAAGAAGGTGGTGAAGCAGG	115 bp
	Reverse	TCAAAGGTGGAGGAGTGGGT	

PCR, polymerase chain reaction.

## Data Availability

The data used to support the findings of this study are available from the corresponding author upon request.
